# OmpK36 GD insertion induces RpoS-dependent *bla*_KPC_ overexpression in carbapenem-resistant *Klebsiella pneumoniae*

**DOI:** 10.1128/aac.01824-25

**Published:** 2026-04-20

**Authors:** Jinnuo Zhu, Yizhen Wang, Changqing Mei, Youhong Weng, Shaoyi Liu, Jianfen Xu, Jiaoli Chen, Jiansheng Huang

**Affiliations:** 1 Department of Clinical Laboratory, Graduate Joint Training Base of Zhejiang Chinese Medical University (Lishui Joint Training Base, Lishui Central Hospital) 70571, Lishui, China; 2 Department of Clinical Laboratory, The Fifth Affiliated Hospital of Wenzhou Medical University, Lishui, China; Entasis, Big Bay, Michigan, USA

**Keywords:** carbapenem resistance, *Klebsiella pneumoniae*, OmpK36, RpoS, stress response, transcriptional regulation

## Abstract

Gly115-Asp116 (GD) insertion in the outer membrane porin OmpK36 is prevalent in carbapenem-resistant, KPC-producing *Klebsiella pneumoniae*, but the molecular mechanisms linking this mutation to enhanced resistance cannot be explained simply by reduced drug permeability. Given that porin mutations compromise bacterial fitness, we investigated the potential role of the stress response regulator RpoS in OmpK36-mediated carbapenemase regulation. Our results demonstrated that among 127 clinical isolates, OmpK36 GD insertion occurred in 50.4% of isolates and was associated with a significantly elevated carbapenem minimum inhibitory concentration and enhanced high-level resistance. In genetically modified strains, the OmpK36 GD insertion impaired bacterial growth and led to a 3.7-fold upregulated *rpoS* expression and a 2.7-fold increase in *bla*_KPC-2_ expression. Bioinformatics analysis identified a putative RpoS binding site in the *bla*_KPC_ promoter region, which was validated by electrophoretic mobility shift assays. Moreover, RpoS overexpression enhanced *bla*_KPC-2_ expression 1.7-fold and increased carbapenem resistance, while disruption of the RpoS binding site reduced *bla*_KPC-2_ expression fourfold and decreased resistance levels. Subinhibitory antibiotic treatment upregulated both *rpoS* and *bla*_KPC-2_ expression across multiple strains, further validating a RpoS-mediated regulatory mechanism. These results suggest that OmpK36 GD insertion contributes to carbapenem resistance through RpoS-mediated transcriptional upregulation of *bla*_KPC_, revealing a stress-activated regulatory mechanism linking porin structural alterations to modulated carbapenemase expression in *K. pneumoniae*.

## INTRODUCTION

Carbapenem-resistant *Klebsiella pneumoniae* (CRKP) represents a major global health threat due to its high mortality and limited treatment options (1, 2). The primary mechanism of carbapenem resistance involves the production of β-lactamase enzymes, particularly *Klebsiella pneumoniae* carbapenemase (KPC), which hydrolyzes nearly all β-lactam antibiotics and accounts for approximately 70% of CRKP isolates worldwide (3, 4). The situation is particularly concerning in China, as CRKP accounts for approximately 90% of carbapenem-resistant *Enterobacteriaceae* isolates (5).

Outer membrane porin mutations, especially the OmpK36 GD insertion, are strongly associated with high-level carbapenem resistance in KPC-producing *K. pneumoniae* (6, 7). Large-scale genomic studies show that OmpK36 loop 3 insertions, including the GD insertion, occur globally in approximately 24% of clinical isolates, increasing to 36% among carbapenemase-producing strains (8). The prevailing view attributes this resistance to reduced porin channel diameter, limiting carbapenem entry into bacterial cells (6, 9). However, studies have shown that different loop 3 insertions, causing varying degrees of pore constriction, can result in similar increases in the carbapenem minimum inhibitory concentration (MIC) (8). Intriguingly, deletion of the KPC-encoding *bla*_KPC_ gene restores carbapenem susceptibility in strains with severe porin defects, indicating that carbapenemase expression remains the key resistance determinant in porin mutants (10). These findings collectively suggest that porin mutations may enhance carbapenem resistance through mechanisms beyond simple pore constriction.

Notably, porin mutations are known to compromise bacterial fitness by restricting nutrient uptake and impairing normal cellular processes (9, 11), potentially activating cellular stress response pathways. RpoS is a global regulatory factor controlling bacterial adaptation to environmental stresses, including nutrient starvation, osmotic stress, and growth inhibition (12–14). In *K. pneumoniae*, RpoS upregulates the expression of genes involved in stress resistance, contributing to enhanced bacterial survival under antibiotic pressure (15). Given that OmpK36 mutations compromise bacterial fitness and considering the central role of RpoS in stress-induced gene regulation, we hypothesized that structural alterations in OmpK36 might activate stress response pathways that could link porin mutations to enhanced carbapenemase expression.

To elucidate the mechanism by which OmpK36 mutations enhance carbapenem resistance in KPC-producing *K. pneumoniae*, we used CRISPR/Cas9 gene editing to create *K. pneumoniae* strains with defined OmpK36 mutations and performed antimicrobial susceptibility testing to evaluate the phenotypic consequences and the effect on RpoS and *bla*_KPC_ expression in the isolates. Furthermore, we performed electrophoretic mobility shift assays (EMSAs), RpoS overexpression experiments, and binding site mutagenesis to evaluate whether RpoS directly binds to and activates the *bla*_KPC_ promoter. Our results demonstrate that OmpK36 GD insertion contributes to carbapenem resistance through RpoS-mediated transcriptional upregulation of *bla*_KPC_, identifying a novel regulatory mechanism linking porin structural alterations to carbapenemase regulation in *K. pneumoniae*.

## RESULTS

### OmpK36 GD insertion is associated with high-level carbapenem resistance

To evaluate the prevalence and phenotypic impact of OmpK36 GD insertion on carbapenem resistance, we collected carbapenem-resistant clinical isolates. Among 127 *K. pneumoniae* clinical isolates harboring the *bla*_KPC_ gene, 64 strains (50.4%) carried the OmpK36 Gly115-Asp116 (GD) insertion; one strain carried a nonsense mutation, and the remaining 62 strains had wild-type OmpK36. The isolates with GD insertions demonstrated significantly higher MICs for the carbapenem imipenem (8 to 128 μg/mL, mean 44.1 ± 28.4 µg/mL) than those with wild-type OmpK36 (2 to 16 μg/mL, mean 6.6 ± 1.9 µg/mL) (*P* < 0.01). The majority of isolates carrying the GD insertion (57/64, 89.0%) exhibited high-level imipenem resistance (MIC ≥32 µg/mL).

To confirm that the OmpK36 GD insertion directly contributed to enhanced resistance, we selected a strain with wild-type OmpK36 (KPN25) and introduced the GD insertion into it using CRISPR/Cas9 editing. The resulting strain, KPN25GD, showed an elevated imipenem MIC (32 μg/mL compared with 8 μg/mL for the parental strain). Next, to assess whether OmpK36 GD insertion alone could account for the observed resistance phenotypes, we eliminated the *bla*_KPC_-carrying plasmids from KPN25 and KPN25GD. Both plasmid-eliminated strains exhibited substantially reduced imipenem MICs (KPN25-Ep and KPN25GD-Ep, 0.063 μg/mL), indicating that KPC expression was essential for carbapenem resistance, regardless of OmpK36 status. Finally, we reintroduced *bla*_KPC_-carrying plasmids into KPN25-Ep and KPN25GD-Ep. Resistance was completely restored in the resulting strains KPN25-Ep-kpc (16 μg/mL) and KPN25GD-Ep-kpc (64 μg/mL). These results demonstrate that carbapenemase expression is the key resistance determinant, even in strains harboring the OmpK36 GD insertion (Table 1, KPN25 strains).

**TABLE 1:** Bacterial strains, plasmids, and MICs of strains used in this study^*a*^

Parental strain	Bacterial strains (*K. pneumoniae*)	Relevant characteristics	Plasmid type	MIC (µg/mL)	
				IPM	MEM
KPN25	KPN25	Wild type	pLSH-KPN25-1	8	16
	KPN25-Ep	Plasmid elimination	N	0.063	0.016
	KPN25-Ep-kpc	KPN25-Ep with reconstructed plasmid	pET28X-kpc	16	32
	KPN25-Ep-mskpc	KPN25-Ep with reconstructed plasmid	pET28X-mskpc	8	16
	KPN25GD	OmpK36 GD insertion	pLSH-KPN25-1	32	32
	KPN25GD-Ep	Plasmid elimination	N	0.063	0.031
	KPN25GD-Ep-kpc	KPN25GD-Ep with reconstructed plasmid	pET28X-kpc	64	128
	KPN25GD-Ep-mskpc	KPN25GD-Ep with reconstructed plasmid	pET28X-mskpc	16	32
KPN18001	KPN18001	OmpK36 GD insertion	pKPN18001-KPC	32	128
	KPN18001-Ep	Plasmid elimination	N	0.063	0.031
	KPN18001GDKO	OmpK36 reversion mutants	pKPN18001-KPC	16	16
	KPN18001KPCKO	*bla*_KPC_ gene knockout	pKPN18001-KPCKO	0.063	0.031
	KPN18001-Ep-kpc	KPN18001-Ep with reconstructed plasmid	pET28X-kpc	64	256
ATCC 13883	ATCC 13883	Standard strain	N	0.008	0.004
	ATCC 13883GD	OmpK36 GD insertion	N	0.016	0.008

^*a*^ IPM, imipenem; KPC, carbapenemase; MEM, meropenem; MIC, minimum inhibitory concentration; MSKPC, KPC with mutated RpoS binding site; N, no plasmid.

For further confirmation, we selected the clinical strain KPN18001 carrying the OmpK36 GD insertion. Similar to the results from KPN25, elimination of *bla*_KPC_-carrying plasmids lowered the imipenem MIC for KPN18001-Ep (0.063 μg/mL) as compared to KPN18001 (32 μg/mL). Moreover, reversion of the OmpK36 GD insertion in the parental strain KPN18001 lowered the resistance levels (16 μg/mL for KPN18001GDKO vs 32 μg/mL for KPN18001), confirming the direct contribution of the OmpK36 GD insertion to carbapenem resistance. Targeted *bla*_KPC_ knockout in strain KPN18001 (KPN18001KPCKO) reduced the imipenem MIC from 32 to 0.063 μg/mL. Finally, resistance was restored and further elevated in KPN18001-Ep-kpc (64 μg/mL) compared with the parental KPN18001 (32 μg/mL) upon transformation with recombinant *bla*_KPC_ constructs. Collectively, these results demonstrate that carbapenemase expression is the key resistance determinant in strains harboring the OmpK36 GD insertion (Table 1, KPN18001 strains).

To evaluate the contribution of OmpK36 GD insertion to carbapenem resistance in the absence of carbapenemase, we also evaluated the effect of OmpK36 GD insertion into the carbapenemase-free strain American Type Culture Collection (ATCC) 13883. Introduction of the OmpK36 GD insertion into ATCC 13883 resulted in only a modest (twofold) increase in the imipenem MIC (0.016 μg/mL for ATCC 13883GD compared with 0.008 μg/mL for ATCC 13883) (Table 1, ATCC 13883 strains). Similar results were observed with meropenem (last column), suggesting that porin alterations promoted resistance, but OmpK36 GD insertion alone was not sufficient to confer significant carbapenem resistance in the absence of carbapenemase.

### OmpK36 GD insertion impairs growth and upregulates *rpoS* and *bla*_KPC-2_ expression

**Fig 1:**
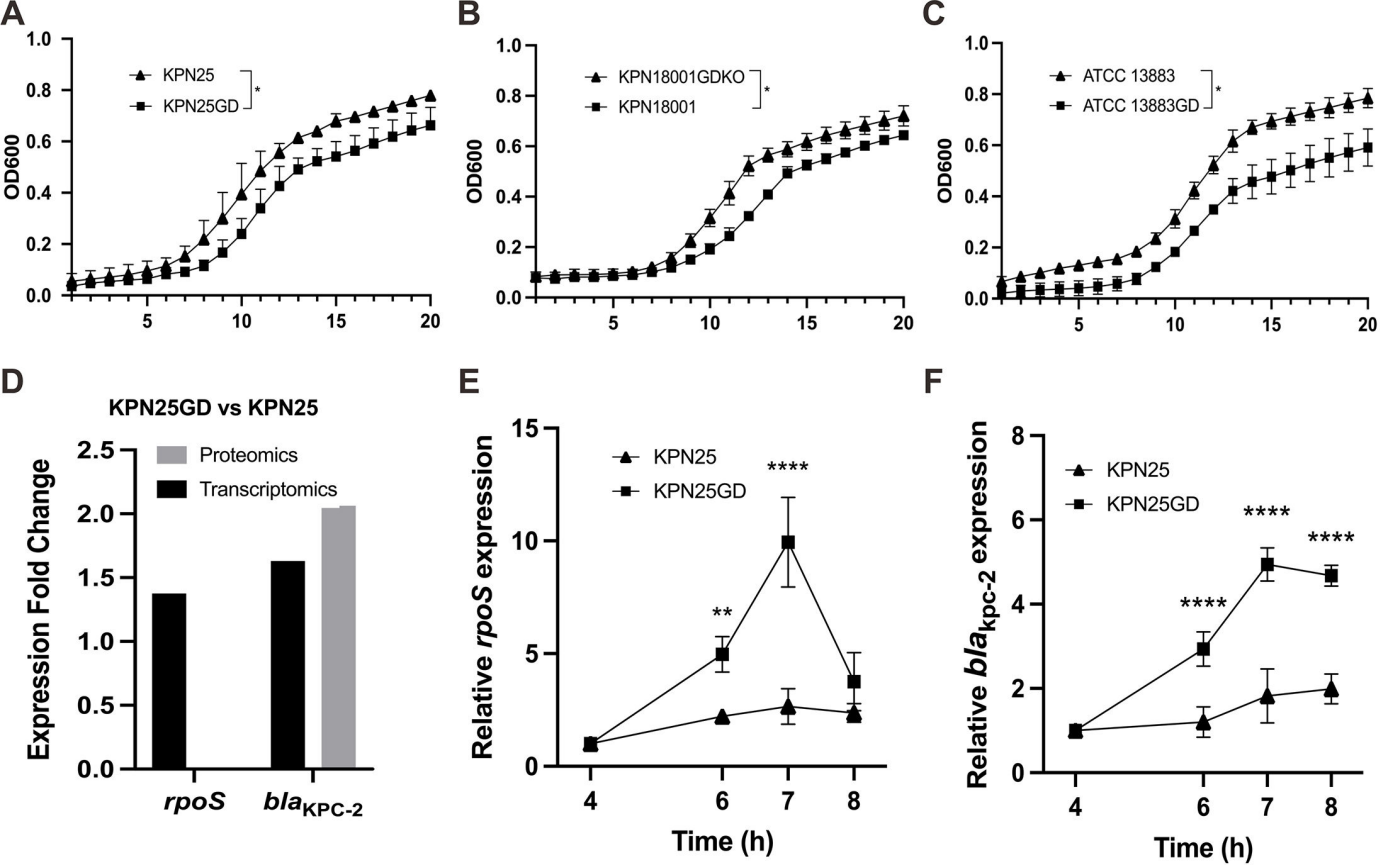
Growth kinetics and gene expression analysis of OmpK36 mutant strains. Growth curves of (**A**) KPN25 (wild-type OmpK36) and KPN25GD (GD insertion), (**B**) KPN18001 (OmpK36 GD insertion) and KPN18001GDKO (GD reversion mutant), and (**C**) ATCC 13883 (wild-type) and ATCC 13883GD (GD insertion), with generation times calculated using G = ln(N2 / N1) / (t2 − t1) × ln (2). Time intervals on the *x*-axis represent 25-min increments. (**D**) Expression levels of *rpoS* and *bla*_KPC-2_ in KPN25GD relative to KPN25, as determined by transcriptomic (black bars) and proteomic (gray bars) analysis. Time-course analysis of (**E**) *rpoS* and (**F**) *bla*_KPC-2_ mRNA expression in KPN25 and KPN25GD strains at 4, 6, 7, and 8 h, normalized to 16S rRNA and all compared with the 4-h time point. Statistical significance: *, *P* < 0.05; **, *P* < 0.01; ****, *P* < 0.0001.

To verify the effect of OmpK36 GD insertion on growth, we performed growth kinetic analysis across multiple strain backgrounds. Strain KPN25GD exhibited longer generation times (0.94 h) compared with the parental strain KPN25 (0.89 h) (*P* < 0.05) (Fig. 1A). Reversion of the GD insertion in clinical strain KPN18001 (KPN18001GDKO) significantly shortened the generation time from 1.44 to 0.97 h (*P* < 0.05) (Fig. 1B). Introduction of the GD insertion into the carbapenemase-free strain ATCC 13883 similarly increased the generation time from 1.05 to 1.21 h (*P* < 0.05) (Fig. 1C). These results indicate that OmpK36 GD insertion consistently impairs bacterial growth across multiple strain backgrounds.

We next examined whether this growth inhibition was associated with changes in *rpoS* and *bla*_KPC-2_ expression through transcriptomic and proteomic analysis of KPN25 and KPN25GD. Transcriptomic analysis revealed that *rpoS* expression was 1.38-fold higher in KPN25GD than in KPN25, while *bla*_KPC-2_ expression was 1.63-fold higher. Proteomic analysis showed more pronounced changes at the protein level, with KPC protein levels increased 2.06-fold in KPN25GD (Fig. 1D). These results indicate that OmpK36 GD insertion upregulates both *rpoS* and *bla*_KPC-2_ at the mRNA level and enhances KPC protein production.

To further characterize the temporal dynamics of this response, we performed time-course quantitative real-time PCR (qRT-PCR) analysis in KPN25 and KPN25GD. Expression differences peaked at 7 h post-inoculation, with *rpoS* (3.7-fold) and *bla*_KPC-2_ (2.7-fold) showing the greatest divergence between KPN25GD and KPN25 (*P* < 0.0001) (Fig. 1E and F). Consistent with these findings, reversion of the GD insertion in clinical strain KPN18001 reduced both *rpoS* and *bla*_KPC-2_ expression at the transcriptional level (Fig. S1).

### RpoS overexpression enhances *bla*_KPC-2_ expression and carbapenem resistance

**Fig 2:**
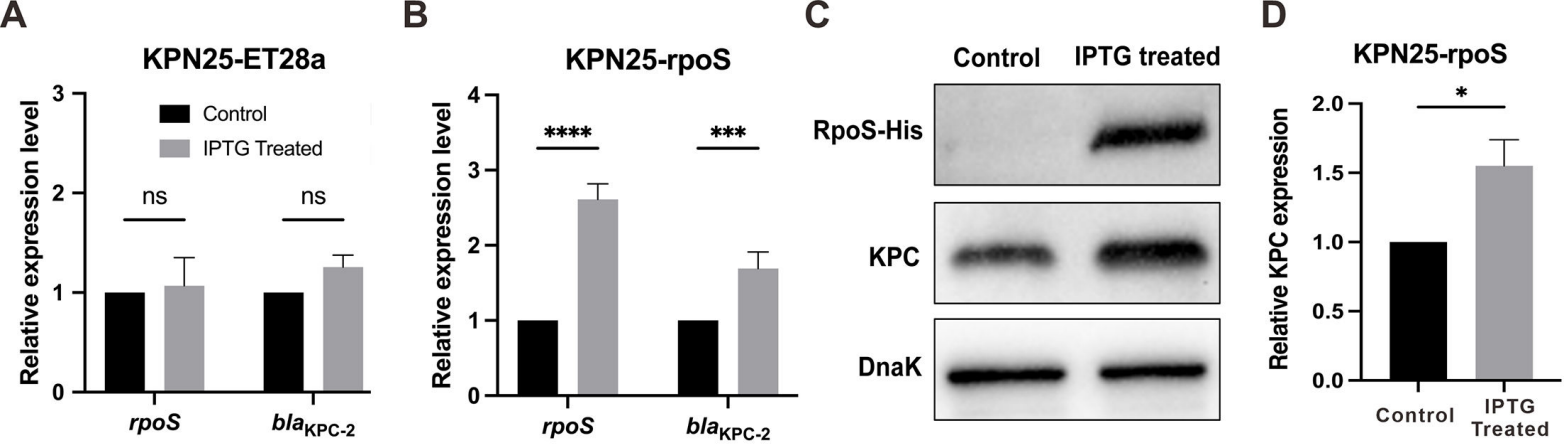
RpoS overexpression and *bla*_KPC-2_ expression analysis. Expression of *rpoS* and *bla*_KPC-2_ in (**A**) KPN25-pET28a (empty vector) and (**B**) KPN25-rpoS strains with or without IPTG treatment, as determined by qRT-PCR. (**C**) Western blot analysis of RpoS and KPC protein production and (**D**) quantitative analysis normalized to DnaK. Control represents strains without IPTG induction. Data are presented as fold change relative to the control, which was set to one for each biological replicate. Error bars represent mean ± SD from three independent experiments. Statistical significance: *, *P* < 0.05; ***, *P* < 0.001; ****, *P* < 0.0001; ns, not significant.

Given that *rpoS* and *bla*_KPC_ are coordinately upregulated in the KPN25GD strain, we hypothesize that RpoS may transcriptionally upregulate *bla*_KPC_ expression. Consequently, we constructed a recombinant strain, KPN25-rpoS, carrying the pET28a-rpoS plasmid, with KPN25-28a as the empty vector control. qRT-PCR analysis revealed that IPTG treatment significantly increased *rpoS* mRNA levels 2.5-fold and *bla*_KPC-2_ mRNA levels 1.7-fold in KPN25-rpoS (*P* < 0.0001 and *P* < 0.001, respectively), while the control strain showed no significant changes upon IPTG treatment (Fig. 2A and B). Western blot analysis confirmed that IPTG treatment significantly increased both RpoS and KPC protein levels in KPN25-rpoS, with KPC protein showing approximately 1.6-fold upregulation compared with untreated KPN25-rpoS cells (*P* < 0.05) (Fig. 2C and D).

To determine whether RpoS overexpression also affects imipenem resistance, we performed MIC assays. After IPTG induction, KPN25-rpoS displayed a twofold increase in imipenem MIC (from 32 to 64 μg/mL), whereas KPN25-28a showed no changes in MICs following induction. Although this shift is reproducible across three independent experiments, the inherent ±2-fold error of the MIC assay warrants cautious interpretation of this result. Nonetheless, the absence of any MIC change in the KPN25-28a control suggests that the observed trend is likely attributable to RpoS-mediated upregulation of KPC expression.

### RpoS binds to the *bla*_KPC_ promoter region

**Fig 3:**
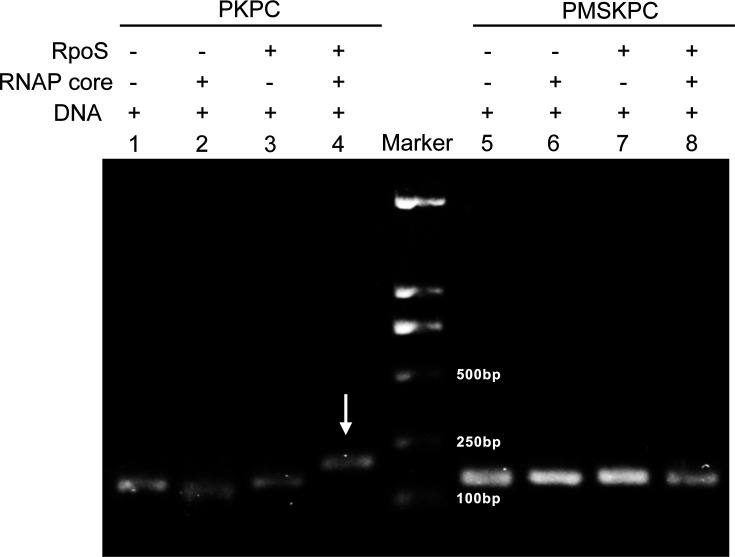
Electrophoretic mobility shift assay of RpoS binding to *bla*_KPC-2_ promoter regions. DNA fragments containing wild-type *bla*_KPC-2_ promoter (PKPC, 162 bp) or mutated RpoS binding sites (PMSKPC, 162 bp) were incubated with purified RpoS protein and/or *E. coli* RNA polymerase core enzyme as indicated. Band retardation occurs with PKPC in the presence of both RpoS and RNA polymerase core enzyme (lane 4, white arrow), while PMSKPC shows no mobility shift (lane 8).

We analyzed the *bla*_KPC_ promoter region using the promoter prediction software BPROM (Softberry) and identified a putative RpoS binding site (TGTCATGA, positions −35 to −28). Consequently, we performed electrophoretic mobility shift assays using purified promoter fragments and reconstituted RNA polymerase complexes to validate this predicted interaction. Neither RpoS protein alone nor RNA polymerase core enzyme alone produced a mobility shift. However, the *bla*_KPC_ promoter fragment (PKPC, 162 bp) showed band retardation when incubated with RpoS-reconstituted RNA polymerase holoenzyme, demonstrating RpoS-dependent promoter recognition. Importantly, a promoter fragment containing a mutated RpoS binding site (PMSKPC) failed to display any mobility shift with the holoenzyme complex (Fig. 3). These results confirm the sequence-specific recognition of the RpoS binding site within the *bla*_KPC_ promoter.

### RpoS binding site mutation reduces *bla*_KPC-2_ expression and carbapenem resistance

**Fig 4:**
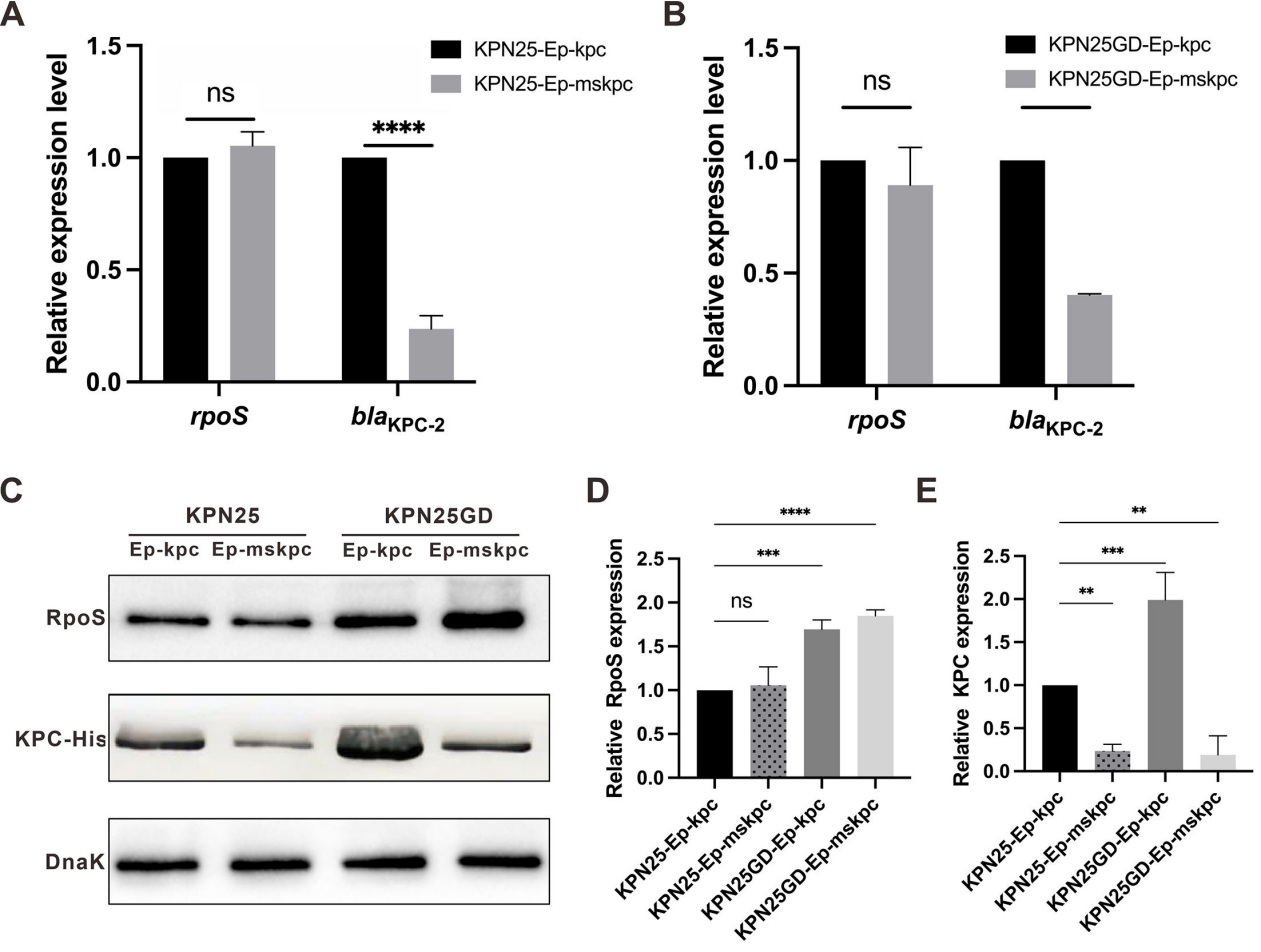
RpoS binding site mutation abolishes *bla*_KPC_ upregulation. Transcriptional levels of rpoS and *bla*_KPC-2_ were measured by qRT-PCR in plasmid-eliminated strains (**A**) KPN25-Ep (wild-type OmpK36) and (**B**) KPN25GD-Ep (GD insertion) after transformation with recombinant plasmids carrying either wild-type *bla*_KPC-2_ promoter (kpc-his, black bars) or mutated RpoS binding site (mskpc-his, gray bars). (**C**) Western blot analysis of RpoS, KPC-His, and DnaK protein levels. Lane 1, KPN25-Ep-kpc; lane 2, KPN25-Ep-mskpc; lane 3, KPN25GD-Ep-kpc; lane 4, KPN25GD-Ep-mskpc. (**D**) Quantitative analysis of relative RpoS protein expression normalized to DnaK across the four strains (KPN25-Ep-kpc, KPN25-Ep-mskpc, KPN25GD-Ep-kpc, and KPN25GD-Ep-mskpc). (**E**) Quantitative analysis of relative KPC protein expression normalized to DnaK across the four strains (KPN25-Ep-kpc, KPN25-Ep-mskpc, KPN25GD-Ep-kpc, and KPN25GD-Ep-mskpc). Data represent mean ± SD from three independent experiments. Statistical significance: **, *P* < 0.01; ***, *P* < 0.001; ****, *P* < 0.0001; ns, not significant.

To evaluate whether the RpoS binding site in the *bla*_KPC-2_ promoter is essential for carbapenemase expression, we performed plasmid-based complementation assays. Plasmid-eliminated strains KPN25-Ep and KPN25GD-Ep were then transformed with recombinant plasmids carrying either the wild-type *bla*_KPC-2_ promoter (pET28X-kpc) or a *bla*_KPC-2_ promoter with a mutated RpoS binding site (pET28X-mskpc). Quantitative RT-PCR analysis after 7 h of cultivation showed that RpoS binding site mutation did not affect *rpoS* expression but significantly reduced *bla*_KPC-2_ mRNA levels. Specifically, *bla*_KPC-2_ mRNA levels were reduced 4-fold in the wild-type OmpK36 background (KPN25-Ep-kpc vs KPN25-Ep-mskpc) and 2.5-fold in the GD insertion background (KPN25GD-Ep-kpc vs KPN25GD-Ep-mskpc) (*P* < 0.0001) (Fig. 4A and B).

To further assess whether these transcriptional changes were reflected at the protein level, we performed Western blot analysis (Fig. 4C). Quantitative analysis showed that KPN25GD-Ep-kpc exhibited 1.7-fold increased RpoS expression compared with KPN25-Ep-kpc (*P* < 0.001), while mutation of the RpoS binding site did not significantly affect RpoS expression in either the wild-type OmpK36 or GD insertion background (Fig. 4D). In contrast, KPC protein expression was twofold higher in KPN25GD-Ep-kpc compared with KPN25-Ep-kpc (*P* < 0.001), and mutation of the RpoS binding site nearly abolished KPC protein expression in both KPN25-Ep-mskpc and KPN25GD-Ep-mskpc, while RpoS levels remained unaffected (Fig. 4E).

Consistent with the reduced *bla*_KPC-2_ expression, antimicrobial susceptibility testing revealed that KPN25-Ep-mskpc and KPN25GD-Ep-mskpc exhibited two- to fourfold lower imipenem and meropenem MICs compared with KPN25-Ep-kpc and KPN25GD-Ep-kpc, respectively (Table 1). Collectively, these results indicate that RpoS binding site mutation reduces *bla*_KPC_ expression without affecting *rpoS* transcription and results in reduced carbapenem resistance.

### Subinhibitory antibiotics stimulate *rpoS* and *bla*_KPC-2_ upregulation

**Fig 5:**
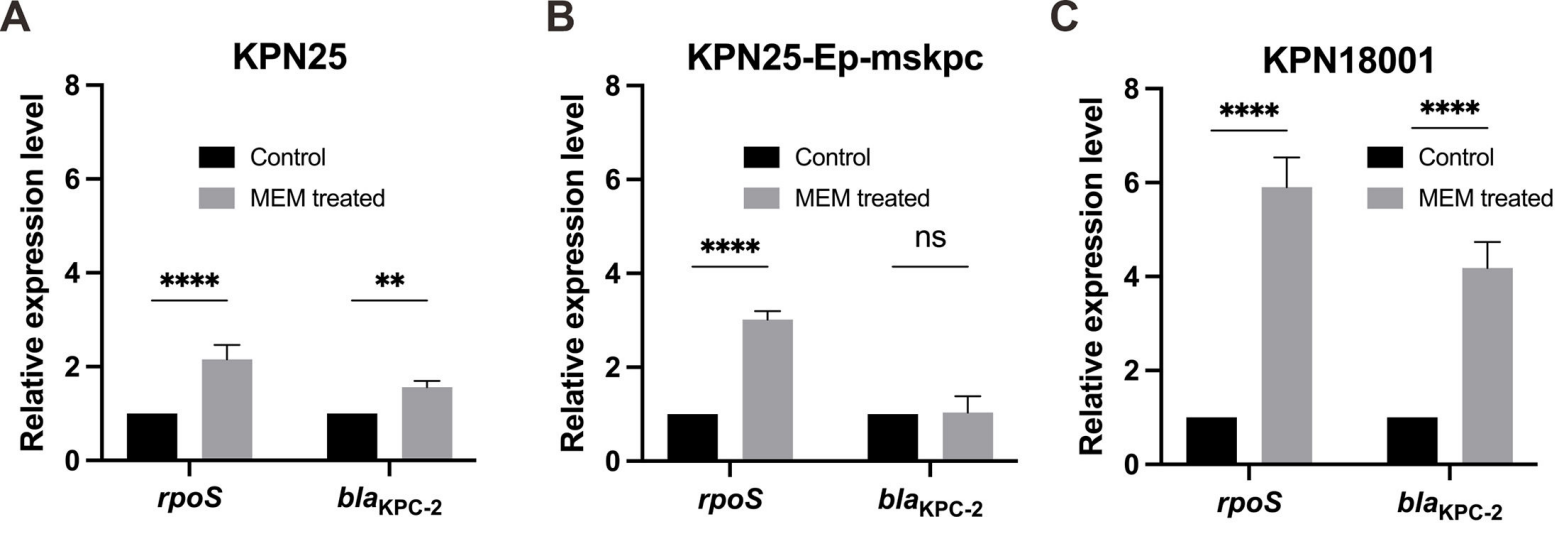
Expression analysis of *rpoS* and *bla*_KPC-2_ upon subinhibitory antibiotic treatment. qRT-PCR analysis of *rpoS* and *bla*_KPC-2_ expression levels in (**A**) KPN25 (wild-type OmpK36), (**B**) KPN25-Ep-mskpc (RpoS binding site mutation), and (**C**) KPN18001 (OmpK36 GD insertion) strains before and after treatment with subinhibitory meropenem concentrations (1/2 MIC for each strain). Statistical significance was determined by two-way ANOVA: **, *P* < 0.01; ****, *P* < 0.0001; ns, not significant.

To determine whether subinhibitory concentrations of meropenem exerted a generalized effect in inducing *rpoS* and *bla*_KPC-2_, we treated KPN25 and KPN18001 with doses equivalent to 1/2 the MIC. Treatment for 7 h significantly elevated *rpoS* expression across all tested strains (KPN25, twofold, *P* < 0.01; KPN18001, sixfold, *P* < 0.0001). Simultaneously, *bla*_KPC-2_ expression was significantly upregulated in KPN25 (1.5-fold, *P* < 0.01) and KPN18001 (4-fold, *P* < 0.0001) (Fig. 5A and C). The RpoS binding site mutant KPN25-Ep-mskpc showed elevated *rpoS* expression (*P* < 0.0001), but *bla*_KPC-2_ expression showed no significant changes (Fig. 5B), confirming the requirement for an intact binding site in antibiotic-induced carbapenemase upregulation. These results demonstrate that antibiotic-induced stress response leads to enhanced KPC enzyme expression via RpoS-mediated transcriptional regulation.

## DISCUSSION

This study identifies a previously unrecognized regulatory mechanism by which OmpK36 GD insertion enhances carbapenem resistance in *K. pneumoniae*. We demonstrate that, beyond the well-established reduction in membrane permeability (16), this porin mutation activates a stress response pathway mediated by the alternative sigma factor RpoS, leading to transcriptional upregulation of *bla*_KPC-2_ and consequently elevated carbapenem resistance. This finding provides a mechanistic explanation for the persistent prevalence of OmpK36 mutations despite their associated fitness costs.

Our analysis of 127 clinical isolates confirmed the high prevalence of OmpK36 GD insertions (50.4%) among carbapenem-resistant *K. pneumoniae* strains. Notably, we established that KPC expression is the fundamental determinant of resistance, as evidenced by the complete restoration of susceptibility upon *bla*_KPC_ deletion or plasmid elimination, even in strains harboring porin mutations. Furthermore, consistent with previous reports (17, 18), we observed that introduction of GD insertion into the carbapenemase-free strain ATCC 13883 produced only a twofold MIC increase. These results demonstrate that carbapenemase production serves as a foundation for OmpK36-mediated resistance, which challenges the conventional view that attributes resistance solely to impaired drug influx.

The observation that OmpK36 GD insertion also imposes significant fitness costs, manifested through extended generation times, provides crucial insight into the physiological consequences of porin mutations. The growth impairment observed in OmpK36 mutant strains underscores the costs of acquiring resistance mutations. Strains with GD insertion showed longer generation times compared to wild-type strains, indicating metabolic stress from porin alterations. This growth defect is consistent with previous reports (6). However, the widespread persistence of GD insertion in clinical isolates suggests that resistance benefits outweigh growth disadvantages under antibiotic selection pressure.

In this study, we demonstrated that growth impairment imposed by the OmpK36 GD insertion creates cellular stress that activates the stress response regulator RpoS. RpoS is a master regulator of the general stress response that controls bacterial adaptation to environmental stresses, including nutrient limitation and growth inhibition (19–22). Transcriptional profiling in OmpK36 mutants revealed upregulation of both *rpoS* and *bla*_KPC-2_. Protein-level analysis confirmed increases in KPC production. Given that RpoS functions as a transcriptional regulator and its upregulation coincides with *bla*_KPC-2_ upregulation, these results suggest that RpoS may directly regulate *bla*_KPC_ expression.

RpoS primarily recognizes the −10 region. Although the −35 region is less critical and more variable for RpoS-dependent promoters (14, 23), our EMSA demonstrated specific binding of the RpoS holoenzyme to the identified site (TGTCATGA), despite its divergence from the consensus sequence in *Escherichia coli*. Additionally, functional validation through RpoS overexpression and binding site mutagenesis confirmed the causal relationship between RpoS activity and *bla*_KPC-2_ expression. Overexpression of RpoS significantly enhanced both *bla*_KPC-2_ transcription and carbapenem resistance, while disruption of the RpoS binding site had the opposite effect. Subinhibitory antibiotic treatment further validated this mechanism by inducing coordinated upregulation of *rpoS* and *bla*_KPC-2_ across multiple strain backgrounds. This response was abolished in strains with a mutated RpoS binding site, confirming the specificity of the pathway. These findings confirm that RpoS-mediated *bla*_KPC-2_ regulation represents a generalized stress response mechanism activated by diverse stimuli, including antibiotic exposure and porin mutation.

The RpoS-mediated regulatory mechanism provides a molecular explanation for the persistence of OmpK36 GD insertion despite the significant fitness cost. The modest but consistent upregulation of carbapenemase production through this stress-activated pathway represents one contributing mechanism by which OmpK36 GD insertion promotes carbapenem resistance. It should be noted that while OmpK36 GD insertion is strongly associated with high-level carbapenem resistance in clinical isolates, the MIC shifts specifically attributable to RpoS-mediated regulation are relatively modest, suggesting that additional factors, such as plasmid copy number, likely cooperate with this pathway to drive high-level resistance. Nevertheless, the identification of this stress-responsive regulatory layer advances our understanding of how porin mutations contribute to the overall resistance phenotype in *K. pneumoniae*. Given the widespread occurrence of OmpK36 mutations in carbapenem-resistant isolates, this stress-activated pathway likely contributes significantly to treatment failures in clinical settings. Further investigation of RpoS-mediated regulatory networks may reveal additional stress-responsive resistance mechanisms in *K. pneumoniae*.

## MATERIALS AND METHODS

### Bacterial strains

The bacterial strains used in this study are shown in Table 1, and the primers are listed in Table S1. A total of 127 non-duplicate carbapenem-resistant *K. pneumoniae* clinical isolates harboring *bla*_KPC_ genes were collected from Lishui Hospital of Zhejiang University between 2021 and 2024. Bacterial identification and plasmid elimination were performed as previously described (4). Representative clinical isolates KPN25 (accession no. CP040391) and KPN18001 (accession no. JBQWBC000000000) were selected for detailed molecular analysis based on their OmpK36 mutation status and carbapenem resistance profiles. *K. pneumoniae* ATCC 13883 was obtained from the American Type Culture Collection and used as a carbapenemase-negative control strain. Strains were routinely cultured in Luria-Bertani (LB) broth or on LB agar plates at 37°C unless otherwise specified.

### Plasmid construction and mutagenesis

RpoS overexpression plasmids and *bla*_KPC_ promoter mutants were constructed using standard molecular cloning techniques. For RpoS overexpression, the *rpoS* gene was amplified from genomic DNA using specific primers (Table S1) and cloned into the pET28a vector (catalog no. A339020; Sangon Biotech, China) between EcoRI and SalI restriction sites. The resulting plasmid pET28a-rpoS was transformed into competent *E. coli* DH5α cells by electroporation and selected on LB agar plates containing 50 μg/mL kanamycin, as described previously (24).

For *bla*_KPC_ promoter analysis, the *bla*_KPC_ gene with its promoter region was amplified and cloned into pET28x, a modified version of pET28a, where the T7 promoter region was deleted using single-primer circular PCR, allowing for the study of native promoter activities without interference from the vector’s promoter. The pET28x vector was constructed as previously described (24, 25). Mutations in the RpoS binding site were introduced using overlap PCR with mutagenic primers (Table S1). The wild-type and mutant constructs were transformed into *E. coli* DH5α and subsequently into *K. pneumoniae* strains through electroporation. Transformants were selected on kanamycin plates and confirmed by PCR amplification and Sanger sequencing. The specific mutation sequences for all constructs are detailed in Table 2.

**TABLE 2:** Mutations constructed in this study^*a*^

Mutation site	Wild-type sequence	Mutant sequence
*ompK36*		
∆GD	GGCGGCGACACC	GGCGGC***GACGGC***GACACC
∆GDKO^*b*^	GGCGGCGACGGCGACACC	GGCGG**CG**ACACC
*bla*_KPC_		
∆KPC	ACCGCGCTGACCAACCTCGTCGCGGAACCATTCGCTAAACTCGAACAGGACTTTGGCGGC	ACCGCG***TAA***GGCGGC
PKPC		
∆MS	GGTTAATGTCATGATAATAAT	GGTTAAT***GGCCGT***ATAATAAT

^*a*^ Underlined and italic nucleotides indicate mutations in each sequence.

^*b*^ The underlined sequence was deleted.

### Plasmid elimination assay

Plasmid elimination was performed using the methods described previously (26). Briefly, bacterial cultures were adjusted to 0.5 McFarland standard and inoculated at a 1:100 ratio into LB broth containing 0.01%–0.04% SDS. Cultures were incubated at 35°C with shaking for 18 h, then subcultured at a 1:100 ratio into fresh LB broth and incubated at 42°C for 22 h. This procedure was repeated, and serial dilutions of the treated cultures were plated on Mueller-Hinton (MH) agar plates. Individual colonies were replica-plated onto MH agar plates containing 4 μg/mL imipenem. Colonies that grew on non-selective MH plates but failed to grow on imipenem-containing plates were selected as potential plasmid-free derivatives. The loss of carbapenemase-carrying plasmids was confirmed by PCR amplification targeting the *bla*_KPC_ gene.

### Generation of CRISPR-Cas9 edited strains

As described previously (27), ompK36 GD loop insertion mutants were constructed in *K. pneumoniae* strains ATCC 13883 and KPN25. Additionally, ompK36 GD loop deletion and *bla*_KPC_ knockout mutants were generated in strain KPN18001. Detailed methods, including sgRNA sequences and primer lists, are provided in the Supplemental material.

### Antimicrobial susceptibility testing

Antimicrobial susceptibility testing was performed using the standard broth microdilution method in cation-adjusted Mueller-Hinton broth according to Clinical and Laboratory Standards Institute (CLSI) guidelines (28). The MICs of imipenem and meropenem were determined for all strains. *Escherichia coli* ATCC 25922 was used as a quality control strain. All tests were performed in triplicate, and results were interpreted according to CLSI breakpoints.

### Bioinformatics analysis of RpoS binding site

The *bla*_KPC_ promoter region (500 bp upstream of the start codon) was analyzed for potential RpoS binding sites using the promoter prediction software BPROM (Softberry, http://www.softberry.com). BPROM predicts bacterial promoters and transcription factor binding sites based on position weight matrices.

### EMSA

DNA fragments containing the *bla*_KPC_ promoter region with wild-type or mutated RpoS binding sites were amplified by PCR using specific primers (Table S1). RNA polymerase core enzyme was purchased from New England Biolabs (catalog no. M0550, Ipswich, MA), and purified RpoS protein was purchased from CUSABIO (catalog no. CSB-EP319548ENVa0; Wuhan, China). For holoenzyme reconstitution, 20 nM *E. coli* RNA polymerase core enzyme and 300 nM RpoS were combined in reconstitution buffer (200 mM Tris-HCl [pH 8.0], 30 mM KCl, 10 mM MgCl_2_, 50 mM NaCl, 1 mM DTT, 1 mM EDTA, and 20 μg/mL BSA) and incubated at 30°C for 45 min.

For binding reactions, purified DNA fragments were incubated with reconstituted RNA polymerase holoenzyme, RNA polymerase core enzyme alone, or RpoS alone in binding buffer (50 mM Tris-HCl [pH 8.0], 200 mM KCl, 3 mM MgCl_2_, 1 mM DTT, 0.1 mM EDTA, 20 μg/mL BSA, and 12 ng/μL poly [dI-dC]) at 25°C for 30 min. Protein-DNA complexes were separated on 1.5% agarose gels and visualized using a Bio-Rad gel imaging system.

### Growth kinetics

Bacterial growth curves were measured using an automated microbial growth curve analyzer. Overnight bacterial cultures were adjusted to 0.5 McFarland standard and diluted 1:200 in fresh LB broth. Samples were incubated at 37°C with shaking at 200 rpm in a Scientz-WSA/A Automated Microbial Growth Curve Analyzer (Scientz, China). Optical density readings were recorded every 5 min throughout the incubation period. Generation times were calculated using the formula *G* = ln(*N*_2_ / *N*_1_) / (*t*_2_ − *t*_1_) × ln (2), where *N*_1_ and *N*_2_ represent optical density values at time points *t*_1_ and *t*_2_, respectively, during the exponential growth phase. All experiments were performed in triplicate.

### qRT-PCR

Transcriptional expression of *rpoS* and *bla*_KPC_ genes was measured by quantitative real-time PCR, as described previously (4). For subinhibitory antibiotic treatment, bacterial cultures were adjusted to 0.5 McFarland standard and grown in LB broth at 37°C with shaking at 200 rpm for 4 h to reach logarithmic phase. Meropenem was then added to achieve a final concentration equivalent to half the MIC for each strain, and cultures were incubated for an additional 7 h before RNA extraction. For IPTG induction experiments, bacterial cultures were grown to logarithmic phase in LB broth containing 1% glucose, then treated with 0.5 mM IPTG for 5 h before RNA extraction. For untreated controls, cultures were grown under identical conditions without antibiotic or IPTG addition.

Total RNA was extracted from bacterial cultures using the TransZol UP RNA kit (catalog no. ER501; TransGen Biotech, China). First-strand cDNA was synthesized using the FastKing One-STep RT-PCR Kit (catalog no. KR118; TIANGEN, China). qRT-PCR analysis was performed on an ABI7500 real-time PCR system (Applied Biosystems, USA) using SYBR Green RT-PCR SuperMix (catalog no. AQ211, TransGen Biotech). The amplification conditions were 95°C for 30 s, followed by 40 cycles of 95°C for 5 s and 60°C for 34 s. Relative gene expression levels were normalized to the 16S rRNA gene using the 2^(−ΔΔCt)^ method. Primers used for qRT-PCR are listed in Table S1. All experiments were performed in triplicate using three independent RNA preparations.

### Western blot

Total proteins were extracted from bacterial cultures using a bacterial total protein extraction kit (catalog no. C600596, Sangon Biotech). For IPTG-induced protein analysis, cultures were treated with 0.5 mM IPTG for 5 h, as described previously, and bacterial pellets were collected for protein extraction. RpoS and KPC protein expression was evaluated by Western blot analysis. Proteins were separated by SDS-PAGE and transferred to nitrocellulose membranes. Membranes were then probed with anti-RpoS antibodies, anti-KPC antibodies, or anti-His-tag antibodies and visualized using a Bio-Rad gel imaging system. Quantitative analysis of Western blot images was performed using ImageJ software. The expression ratio of target protein to the housekeeping protein DnaK was calculated to compare differences between experimental groups. All experiments were performed in triplicate, and results were normalized to DnaK expression levels.

## SUPPLEMENTAL MATERIAL

Supplemental material aac.01824-25-s0001.docx Fig. S1; Tables S1 and S2.

ASM does not own the copyrights to Supplemental Material that may be linked to, or accessed through, an article. The authors have granted ASM a non-exclusive, world-wide license to publish the Supplemental Material files. Please contact the corresponding author directly for reuse.
