# Supplementary material for: OmpK36 GD insertion induces RpoS-dependent blaKPC overexpression in carbapenem-resistant Klebsiella pneumoniae
Source: Antimicrob Agents Chemother. 2026 Apr 20;70(6):e01824-25. doi: 10.1128/aac.01824-25 (PMC13231924; doi:10.1128/aac.01824-25)
Supplement: Supplemental material — Fig. S1; Tables S1 and S2. [file aac.01824-25-s0001.docx]

**Supplementary materials**

**
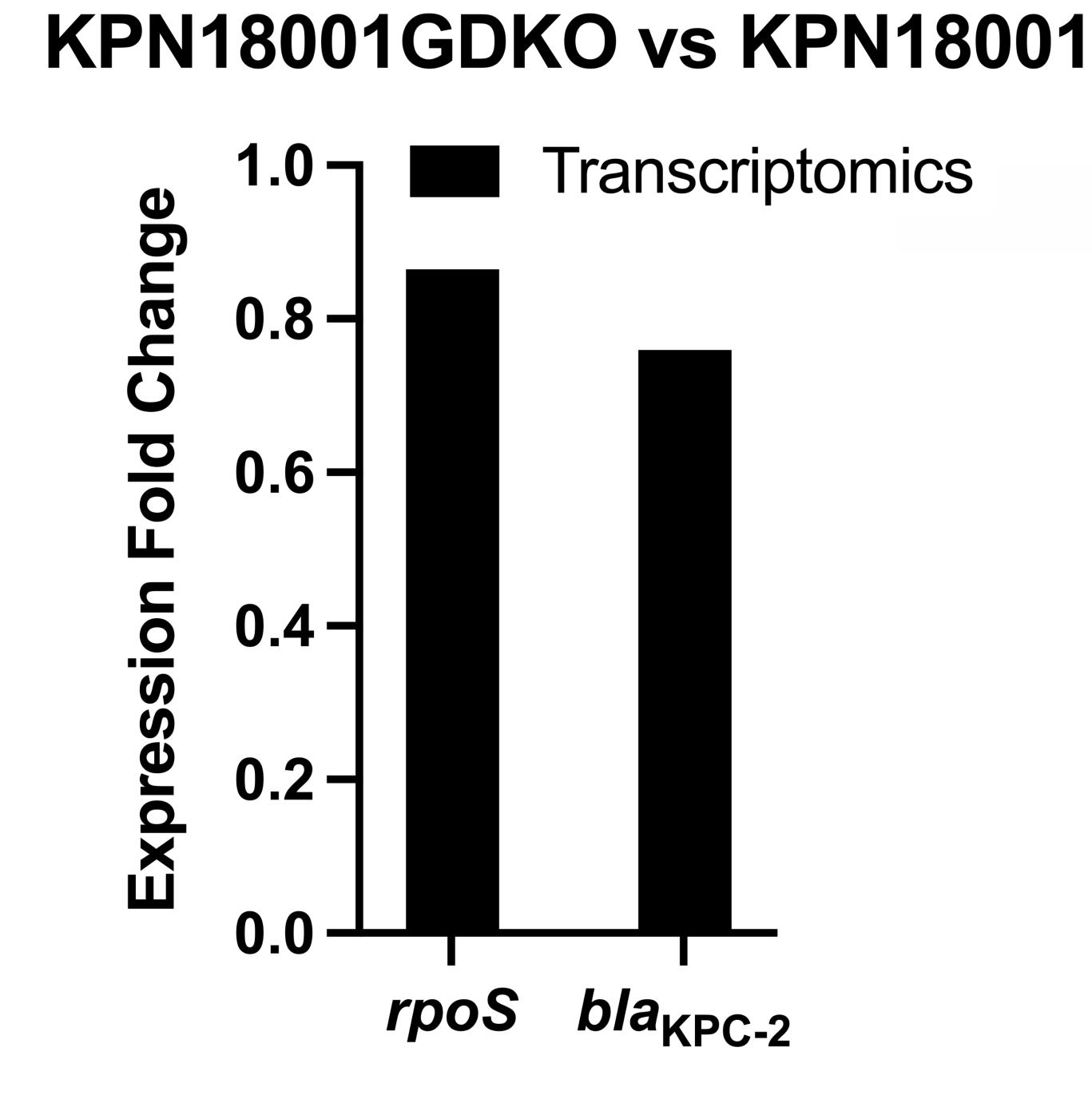
**

**Figure S1** Gene expression analysis of *rpoS* and *bla*_KPC-2_ in KPN18001 and KPN18001GDKO. Expression levels of *rpoS* and *bla*_KPC-2_ in KPN18001GDKO relative to KPN18001, as determined by transcriptomic analysis (black bars).

**Generation of CRISPR-Cas9 Edited Strains**

**Bacterial strains and plasmids.**

The study utilized three parental *K. pneumoniae* strains: KPN25 and ATCC 13883 (harboring a natural ompK36), and KPN18001 (harboring the mutant ompK36 with a GD insertion and *bla*_KPC_). Genetic manipulations employed the CRISPR-Cas9 system using the temperature-sensitive plasmid pKCas9 and sgRNA-expressing plasmids pSGKP or pTcsgRNA (1).

**Construction of sgRNA plasmids.**

Specific 20-nt sgRNA sequences were designed and cloned into BsaI-digested vectors to target strain-specific alleles (Table S2):

For GD loop insertion (in KPN25/ATCC 13883): The identical sgRNA sequence 5'-GAATTCGGCGGCGACACCTA-3' was used for both strains. This sgRNA specifically targets the unique junction sequence formed by the natural 6-bp deletion, ensuring precise cleavage of the GD-deficient allele while sparing the restored wild-type sequence after editing. For GD loop deletion (in KPN18001): The sgRNA 5'-GGCGGCGACGGCGACACCTA-3' was designed to target the coding region spanning the GD loop in the intact ompK36 allele, inducing a double-strand break to facilitate replacement with a GD-deleted donor template. For *bla*_KPC_ knockout (in KPN18001): The sgRNA 5'-GAATGGTTCCGCGACGAGGT-3' targeted the coding region of *bla*_KPC_ to facilitate the introduction of a premature stop codon. All oligonucleotides were annealed, ligated into vectors, and verified by Sanger sequencing.

**Preparation of homologous recombination templates.**

Linear DNA fragments for homology-directed repair (HDR) were prepared as follows: GD Loop insertion: Donors containing the intact 6-bp GD sequence (GGCGAC) flanked by ~500 bp homologous arms were generated via overlap extension PCR using mutagenic primers (Table S2). GD Loop Deletion: A donor fragment spanning the GD insertion site but excluding the 6-bp GD codons was amplified from ATCC 13883 genomic DNA using primers 18001-13883-GD-KZ-F/18001-13883-GD-KZ-R (Table S2), as the sequence at this locus in ATCC 13883 is identical to the wild-type OmpK36 sequence targeted for deletion. *bla*_KPC_ Knockout: A repair template containing a premature stop codon (taa) replacing the critical catalytic site codons was constructed via overlap extension PCR using mutagenic primers 18001-KPC-mut-F and 18001-KPC-mut-UP-R.

**Electroporation and strain selection.**

Electrocompetent cells harboring pKCas9 were co-transformed with the specific sgRNA plasmid and the corresponding linear HDR template (~500 ng). Transformants were selected on LB agar with kanamycin and spectinomycin at 30°C. Colonies were screened by colony PCR and Sanger sequencing:

**Curing of plasmids and verification.**

Plasmids were cured by incubation at 42°C. Final mutants were verified by Sanger sequencing of the target loci to ensure precise edits and the absence of off-target mutations.

**Table S1** Primers used in this study

| Primers |  |
| --- | --- |
| Primers for gene amplification |  |
| ompK36-F | TAGCCGACTGATTAGAAGGG |
| ompK36-R | CAAGAGTATACCAGCGAGGT |
| KPC-F | GCTACACCTAGCTCCACCTTC |
| KPC-R | ACAGTGGTTGGTAATCCATGC |
| E8F | CAGTACGAATTCGCTTCAACGGTCGTATCAGCGCTTCAACGGTCGTATC |
| KPHISR | GCAAGCTTGTCGACCTGCCCGTTGACGCCCAATC |
| RpoSF | GGTCGCGAATTCGGTATCGATATTGCAGGCAG |
| RpoS-F2: | GGTCGCGAATTCAGTCAGAATACGCTG |
| RpoSR | GGCCGCAAGCTTGTCGACTTCGCGGAAGAGCGCTTCGA |
| rpoS-bsF | AAACACCCGTTCAGGCGC |
| rpoS-bsR | GGGTTCCGCGCACATTTC |
| MCS-F: | ACGGAGCTCGAATTCGGATC |
| MCS-R: | ACGTAGGTCGACAAGCTTGCGGCCGC |
|  |  |
| Primers for qRT-PCR |  |
| qKPC-F | GGCGGCTCCATCGGTGTGTA |
| qKPC-R | AATTGGCGGCGGCGTTATCA |
| qRPOS-F | GCGATCTGGCTGAGGAAGAG |
| qRPOS-R | GCTGTAACGACGGGCAATCT |

**Table S2** Primers and sgRNA sequences used for genome editing

| **Name** | **Sequence (5'-3')** | **Purpose** | **Target/Strain** |
| --- | --- | --- | --- |
| GD Loop insertion  (in KPN25/13883) |  |  |  |
| sgRNA-25/13883-GD | GAATTCGGCGGCGACACCTA | Cleavage of GD-deficient allele | KPN25,13883 |
| KP25-GD-Mut-F | GCGGCgacggcGACACCTACGGTTCTGACAACTT | Introduces GD codons | KPN25 |
| KP25-GD-Mut-R | TGTCgccgtcGCCGCCGAATTCCGGCAGAACGT | Introduces GD codons | KPN25 |
| 13883-GD-Mut-F | GCGGCgacggcGACACCTACGGTTCTGACAACTT | Introduces GD codons | ATCC 13883 |
| 13883-GD-Mut-R | TGTCgccgtcGCCGCCGAATTCCGGCAGAACGT | Introduces GD codons | ATCC 13883 |
| GD Loop Deletion  (in KPN18001) |  |  |  |
| sgRNA-18001-GDKO | GGCGGCGACGGCGACACCTA | Cleavage of GD-containingallele | KPN18001 |
| 18001-13883-GD-KZ-F | ACTGAAAGCTCCAGCGATCAGG | Repair template amplification / Colony PCR screening | ATCC 13883/  KPN18001 |
| 18001-13883-GD-KZ-R | GGTGTACTGAGTCGCCAGGTA | Repair template amplification /  Colony PCR screening | ATCC 13883/  KPN18001 |
| *bla*_KPC_ Knockout  (in KPN18001) |  |  |  |
| sgRNA-KPC-KO | GAATGGTTCCGCGACGAGGT | Cleavage of *bla*_KPC_ | KPN18001 |
| 18001-KPC-mut-F | CTTTTCTGCCACCGCGtaaGGCGGCTCCATCGGTGTGTAC | Introduces Stop Codon | KPN18001 |
| 18001-KPC-mut-UP-R | CCGATGGAGCCGCCttaCGCGGTGGCAGAAAAGCCA | Introduces Stop Codon | KPN18001 |

**Reference**

1. Wang Y, Wang S, Chen W, Song L, Zhang Y, Shen Z, Yu F, Li M, Ji Q. 2018. CRISPR-Cas9 and CRISPR-Assisted Cytidine Deaminase Enable Precise and Efficient Genome Editing in Klebsiella pneumoniae. Appl Environ Microbiol，2018. **84**(23): p. e01834-18.
